# Rupture d’un anévrisme de l’artère splénique en fin de grossesse: à propos d’un cas

**DOI:** 10.11604/pamj.2019.34.63.18598

**Published:** 2019-10-01

**Authors:** Ahmed Tlili, Aymen Trigui, Oussema Dkhil, Wiem Feki, Haithem Rejab, Hazem Ben Ameur, Salah Boujelbene, Zeineb Mnif

**Affiliations:** 1 Faculté de Médecine de Sfax, Service de Chirurgie Viscérale et Générale, Hôpital Habib Bourguiba, Sfax, Tunisie; 2 Faculté de Médecine de Sfax, Service d’Imagerie Médicale, Hôpital Hedi Chaker, Sfax, Tunisie

**Keywords:** Anévrisme, rupture, artère splénique, grossesse, mort fœtal in utéro, Aneurysm, rupture, splenic artery, pregnancy, fetal death in utero

## Abstract

Les hémorragies lors de la grossesse peuvent être d'origine non obstétricale, la grossesse étant un terrain favorisant pour certaines étiologies du fait des modifications physiologiques qu'elle induit. Ces hémorragies non obstétricales sont rares mais sont responsables d'une mortalité materno-fœtale importante. Le pronostic dépend de la rapidité du diagnostic et d'une prise en charge multidisciplinaire. La rupture per gravidique d'un anévrysme de l'artère splénique (AAS) est une affection rare mais de pronostic redoutable. Le tableau clinique typique associant douleur abdominale, hypotension et anémie est très trompeur pour l'obstétricien qui évoque plus volontiers un hématome rétro placentaire ou une rupture utérine. Nous rapportant le cas d'une patiente enceinte ayant nécessité une laparotomie en urgence devant la découverte à l'imagerie d'une rupture d'anévrysme de l'artère splénique.

## Abstract (en)

Bleeding during pregnancy may not be due to obstetric causes. Pregnancy is a predisposing factor for some disorders due to physiological changes. These obstetric bleedings are rare but are responsible for high materno-fetal mortality. Prognosis depends on the speed of diagnosis as well as on multidisciplinary management. Splenic artery aneurysm (SAA) rupture during pregnancy is rare with a dreadful prognosis. Common clinical signs including abdominal pain associated with hypotension and anemia are very misleading for the obstetrician who usually suspects retroplacental hematoma or uterine rupture. We report the case of a pregnant patient requiring emergency laparotomy due to the detection of splenic artery aneurysm rupture on imaging test.

## Introduction

Bien que plus rares que les hémorragies du post-partum, de nombreux cas d'hémorragies intra péritonéales ont été décrits chez la femme enceinte, la grossesse étant un terrain favorisant pour certaines étiologies comme la rupture d'un anévrysme artériel splénique qui est une complication rare et redoutable mais peu prévisible. La survie materno-fœtale dépend de la rapidité diagnostique, de la qualité du geste d'hémostase et du retentissement fœtal.

## Patient et observation

Il s'agit d'une patiente âgée de 37 ans, 2^ème^ pare, sans antécédents pathologiques notables, bien suivie pour une grossesse, consulte aux urgences gynécologique pour dyspnée, douleur abdominale diffuse d'installation brutale sur une grossesse à terme (39 semaines d'aménorrhée). À l'examen, versant maternel: patiente très algique, sueur profuse, tension artérielle de 11/7 mmHg, fréquence cardiaque 100 bpm, défense abdominale généralisée, abdomen très distendu avec une hauteur utérine concordante avec le terme, pas de saignement vaginal; à la biologie: hémoglobine 9 g/dl, hypoxémie et hypocapnie au gaz du sang, le reste du bilan est sans anomalies notamment la lipasémie et le bilan hépatique; versant fœtal: le diagnostic de mort fœtal in utéro devant, l'absence des mouvements actifs fœtaux depuis 24 heures négligé par la patiente, un tracé du rythme cardiaque fœtal plat et devant l'absence d'activité cardiaque à l'échographie.

**Figure 1:**
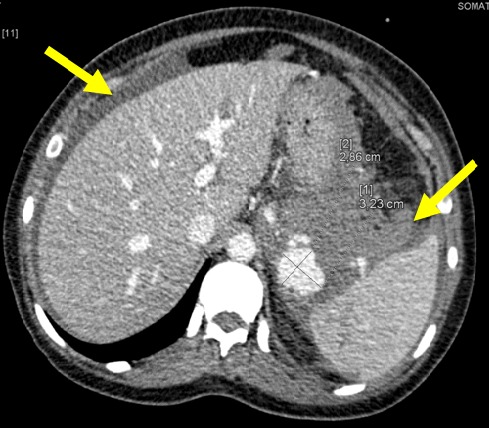
Épanchement liquidien intra péritonéal de grande abondance

**Figure 2:**
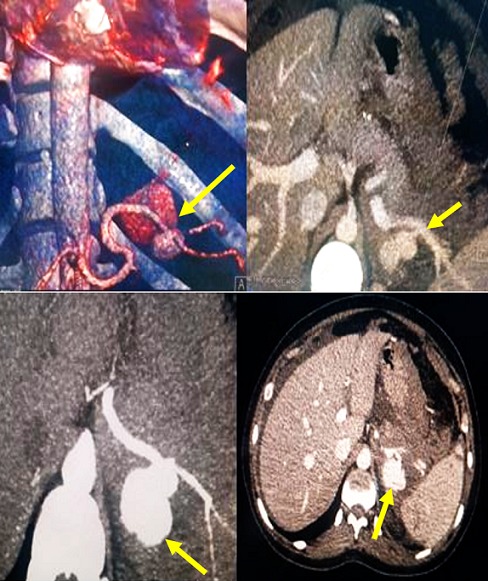
Anévrysme sacciforme de l´artère splénique proximale mesurant 3 cm de grand axe et 4 mm au niveau de son collet, avec mise en évidence d´une extravasation du produit de contraste à ce niveau se présentant comme une flaque spontanément hyperdense

**Figure 3:**
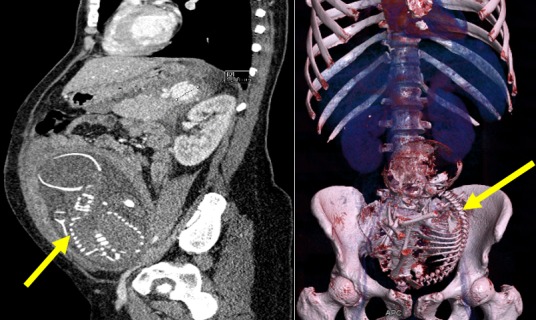
Mort fœtal in utéro objectivée par l´absence de rehaussement du fœtus ni du versant fœtal du placenta

Un angioscanner thoracique a été demandé en urgence afin d'éliminer une embolie pulmonaire associée à un scanner abdominal dans un but étiologique. À l'imagerie on n'a pas objectivé une embolie pulmonaire ni un foyer de pneumopathie à l'étage thoracique. À l'étage abdominal on a noté: un pancréas de taille normale ayant conservé ses lobulations; épanchement liquidien intra péritonéal de grande abondance avec mise en évidence d'un épanchement dense en regard du hile splénique en péri splénique en inter anse et au niveau de la gouttière pariéto-colique gauche (Figure 1). Après injection du produit de contraste on a noté la présence d'un anévrysme sacciforme de l'artère splénique proximale mesurant 3 cm de grand axe et 4 mm au niveau de son collet. Il est de contours irréguliers flous par endroits avec mise en évidence d'une extravasation du produit de contraste à ce niveau se présentant comme une flaque spontanément hyperdense. Il est situé à 9 cm de l'origine de l'artère splénique et à 4.5 cm du hile splénique (Figure 2). Il s'y associe un deuxième anévrysme sacciforme contigu situé en aval à celui précédemment décrit mesurant 18 mm de grand axe et 4 mm au niveau de son collet (Figure 2). Multiples foyers d'infarctus splénique (Figure 2); mort fœtal in utéro objectivé par l'absence de rehaussement du fœtus ni du versant fœtal du placenta (Figure 3).

Une heure après l'examen, la patiente a eu un état de choc hémorragique (tension artérielle 8/5 mmHg, fréquence cardiaque à 120 bpm) avec hémoglobine de contrôle à 6 g/dl. Elle a bénéficié d'un remplissage vasculaire 1000 ml de Voluven et d'une transfusion sanguine et après une brève réanimation la patiente a eu une laparotomie médiane objectivant un hémo péritoine de grande abondance évalué à 1200 ml provenant d'un anévrysme rompu de l'artère splénique. La patiente a bénéficiée d'une splénectomie avec pancréatectomie caudale emportant les deux anévrysmes associés à une césarienne. Bonne évolution par la suite avec stabilisation hémodynamique et la sortie a été autorisée après 10 jours de l'opération. À noter que la patiente a été proposée pour embolisation artérielle qui n'était pas faite par défaut de matériel (coil).

## Discussion

La rupture d'anévrysme de l'artère splénique en cours de grossesse est une affection rare mais de pronostic redoutable. En 1993, Angelakis *et al.* [1, 2] rapportait une mortalité maternelle à 75%, une mortalité fœtale à 90%. L'incidence de l'anévrysme de l'artère splénique dans la population générale est estimée à moins de 1% [1, 2] mais s'élève avec l'âge pour dépasser les 10% après 60 ans. La grossesse contribue à l'apparition et/ou à la rupture de l'anévrysme par 2 mécanismes: la fragilisation des parois vasculaires liée à un processus dégénératif gravidique (dysplasie des fibres de la média, dédoublement et rupture de la lame élastique interne, fragmentation des fibres élastiques) [1-3]; l'hypertension portale induite par l'augmentation du volume utérin. Cette association “hypertension-fragilisation vasculaire” peut être aggravée par une anomalie congénitale du tissu conjonctif et par l'augmentation du débit vasculaire splénique au cours de la grossesse. D'autres localisations vasculaires sont affectées de la même manière comme le montrent l'augmentation du risque de dissection aortique (50% des dissections aortiques chez la femme de moins de 40 ans surviennent en cours de grossesse [4, 5]), la formation d'anévrysmes de l'aorte sous rénale, des axes iliaques, de l'artère hépatique ou de la mésentérique supérieure. Devant la découverte d'un anévrysme, il est donc nécessaire de rechercher d'autres sites anévrysmaux associés.

Quatre-vingt-quinze pour cent des anévrysmes de l'artère splénique restent asymptomatiques jusqu'à leur rupture [6]. Des symptômes peuvent apparaître dans 5 à 20% des cas: il s'agit de douleurs intermittentes localisées à l'épigastre ou à l'hypochondre gauche, de nausées voire de vomissements liés à l'augmentation du volume de l'anévrysme. On peut percevoir un souffle vasculaire ou palper une splénomégalie voire l'anévrysme lui-même [6]. La découverte peut être fortuite au cours d'un examen échographique ou d'un cliché d'abdomen sans préparation (70% des anévrysmes de l'artère splénique sont calcifiés et se révèlent par le signe de “l'anneau” sur les radiographies [6]) ou lors de la complication la plus grave qui est la rupture spontanée. Celle-ci survient généralement au 3^ème^ trimestre (70% des cas) et de manière brutale (75% des cas). La patiente présente une douleur de l'hypochondre gauche en coup de poignard, voire un signe de Kehr, puis rapidement étendue à l'ensemble de l'abdomen. Un choc hémorragique s'installe plus ou moins vite en fonction du volume de l'hémopéritoine. La souffrance fœtale aiguë devant le choc maternel car les mécanismes d'adaptation hémodynamique réduisent précocement la perfusion placentaire (cas de notre observation en présence d'une souffrance fœtale alors que l'hémodynamique maternelle restait stable). Cette chronologie défavorable au fœtus explique la surmortalité fœtale (90% des cas). La rupture peut avoir lieu en 2 temps (25% des cas). Dans cette situation, l'hémopéritoine reste localisé à la bourse rétroépiploïque puis gagne secondairement la grande cavité péritonéale à travers le foramen de Winslow ou par d'autres voies (exemple: à travers le méso colon transverse). L'intervalle libre est de durée variable et sa symptomatologie n'est pas toujours présente. Même en présence d'un tableau typique de rupture anévrysmale associant douleur abdominale, hypotension artérielle et anémie, la rupture reste souvent mal diagnostiquée car sa présentation montre des symptômes presque identiques à ceux des urgences obstétricales courantes, telles que le décollement du placenta, la rupture utérine, une embolisation liquidienne amniotique et dans les grossesses précoces, la rupture d'une grossesse extra-utérine [7]. La méconnaissance de cette affection, induit régulièrement un retard à la prise en charge, source de surmortalité.

Lorsque l'exploration chirurgicale d'un hémopéritoine en cours de grossesse révèle un saignement d'origine splénique, il faut réaliser une splénectomie. Essayer de pratiquer la ligature sélective des vaisseaux anévrysmaux expose à une dissection délicate et dangereuse (environ 80% des anévrysmes se trouvent sur le tiers distal de l'artère [8] obligeant la dissection du hile); l'urgence du tableau clinique ne tolère pas une telle perte de temps [9]. Si un anévrysme de l'artère splénique est découvert de façon fortuite (au cours d'une radiographie de l'abdomen ou d'une échographie) ou sur signes d'appel chez une femme en âge de procréer ou enceinte, il faut recourir au traitement chirurgical préventif. Une embolisation artérielle ou une excision chirurgicale de l'anévrisme est recommandée si la patiente est en âge de procréer ou si l'anévrisme a un diamètre supérieur à 2 cm [10, 11]. Des techniques chirurgicales peu invasives acceptées pour traiter les SAA consistent en une ligature de l'artère splénique, une embolisation artérielle, une résection de l'artère splénique et une splénectomie [7]. Les techniques d'embolisation peuvent être tentées pour tous les anévrysmes de l'artère splénique, à l'exception de ceux situés près du hile de la rate et chez les patientes enceintes en raison du risque d'exposition aux rayonnements. Cependant, le suivi à long terme de ce traitement n'est pas disponible et son taux de réussite est inférieur à 85% pour des raisons techniques liées à l'anatomie de l'anévrisme [12, 13]. Les complications de cette technique incluent la migration de la spirale et l'infarctus distal, la formation d'abcès, la rupture de l'anévrysme et la recanalisation chez 12,5% des patients après une embolisation réussie.

## Conclusion

La rupture d'anévrysme splénique per gravidique est de pronostic redoutable. La rareté de sa survenue et sa révélation cliniquement trompeuse pour l'obstétricien rendent le diagnostic trop souvent tardif. Les adaptations hémodynamiques maternelles réduisent précocement la perfusion placentaire occasionnant une souffrance fœtale aiguë avant la survenue du choc hémorragique maternel. La laparotomie exploratrice en urgence, de préférence médiane, par un chirurgien expérimenté, permet la réalisation conjointe de l'extraction fœtale et de la splénectomie d'hémostase.

## Conflits d’intérêts

Les auteurs ne déclarent aucun conflit d'intérêts.
